# Investigation of differences in mechanisms of die filling between a compaction simulator and a rotary press

**DOI:** 10.1016/j.ijpx.2025.100405

**Published:** 2025-09-23

**Authors:** Ben Kohlhaas, Jan Henrik Finke

**Affiliations:** a Institute for Particle Technology, Technische Universität Braunschweig, Volkmaroder Straße 5, 38104 Braunschweig, Germany; b Center of Pharmaceutical Engineering (PVZ), Technische Universität Braunschweig, Franz-Liszt-Straße 35A, 38106 Braunschweig, Germany

**Keywords:** Tableting, Die filling, Rotary tablet press, Compaction simulator, Suction filling, Flowability, Process transfer

## Abstract

Die Filling is the critical process step in tableting as it determines the tablet weight and its variability as well as impacting tablet strength and defect propensity. Several studies have focused on modeling die filling on rotary presses, however none have investigated the matter on a compaction simulator. Therefore, the aim of this study is to characterize the die filling behavior on a compaction simulator and compare it to a laboratory scale rotary press. Special attention is paid to the complex interplay of process parameters, machine geometry and material properties. Experimental results are supported by a newly introduced physics-based calculation of the course of the exerted differential pressure as a main driver of die filling. On the compaction simulator, suction filling is shown to be more intense due to its geometry and elevated lower punch velocities, rendering paddle speed of the feed frame less crucial. On the rotary press, paddle rotation is necessary to ensure sufficient powder flow into the dies, especially at high production speed, due to a shorter filling time. An alternative fill cam geometry, where the punch is already pulled down to a certain extent before entering the feed frame, reduces the exerted suction pressure in the filling zone, giving generally lower filling yield for materials of limited flowability. The study offers a solid understanding of die filling on a compaction simulator and the underlying mechanisms. Together with the comparative experiments, the foundation for a model for rational scale transfer towards rotary presses is established.

## Highlights

• first investigation of the degree of die filling on a compaction simulator.
• interplay of dynamic conditions, machine geometry and powder properties is studied.
• newly introduced numerical calculation provides insight into filling mechanisms.
• suction filling is the main driver of die filling on the compaction simulator.

## Graphical Abstract

**Figure:**
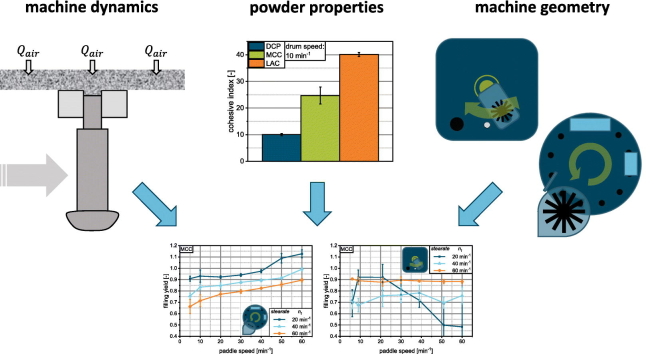

## 1 Introduction

Among oral pharmaceutical dosage forms, the tablet is the most widely applied due to its low manufacturing costs and high acceptance by patients. Consequently, considerable resources are attributed to formulation and process development of tablets in the pharmaceutical industry. Especially in early formulation development, where only little material is available, compaction simulators are widely used studying the compaction, ejection and take-off behavior of formulation candidates (Desbois et al., 2019; Mazel et al., 2019; Schönfeld et al., 2024; Sun, 2015; Wang et al., 2004). However, the sub-process of die filling is only poorly understood on compaction simulators, mostly covering the impact of the feeding process on the formulation itself. Wünsch et al. studied the filling stage with regard to lubricant dispersion and the resulting drop in tablet tensile strength due to higher shearing intensity in the feed frame of the compaction simulator when compared to a rotary press (Wünsch et al., 2020). Puckhaber et al. continued this work by developing a model to predict the effect of lubricant dispersion on compressibility and compactibility (Puckhaber et al., 2024; Puckhaber et al., 2023b; Puckhaber et al., 2023a; Puckhaber et al., 2022b; Puckhaber et al., 2022a). However, the degree of die filling itself was not systematically investigated. This poor understanding is in stark contrast to the production scale, where die filling is considered the crucial step, as it not only determines tablet weight and its consistency, but also impacts tablet strength and defect propensity by over- or underfilling. If issues with die filling arise during scale up of a formulation from pilot plant to production scale, expensive trials on the respective machines have to be conducted to optimize the process conditions, the worst-case scenario being the necessity for changes to the formulation, causing significant costs due to the associated regulatory process.

To mitigate this risk, Schomberg et al. have developed a model to describe die filling under gravity filling conditions on a laboratory scale rotary press (Schomberg et al., 2023b). In gravity filling, the punch enters the filling are already pulled down (in the most drastic cases already to the lowest punch position of the fill cam before dosing out). The powder flows into the dies by means of the gravitational force, usually aided by the rotation of the paddle wheel supplying sufficient powder flow to the filling zone. The model predicts the critical paddle speed to achieve complete die filling at a given turret speed and requires only simple material properties such as the bulk density and permeability. It was consecutively extended towards production scale rotary presses, allowing for a rational scale transfer based on material and process parameters (Schomberg et al., 2023a).

Gravity filling has also been extensively studied on linear filling devices with a moving feed shoe and stationary die (Baserinia and Sinka, 2019; Schneider et al., 2007; Sinka et al., 2009; Sinka et al., 2004; Wu et al., 2003). Zakhvatayeva et al. studied gravity filling on a rotary test rig to evaluate the transferability of these results towards rotary presses (Zakhvatayeva et al., 2018). It was found that powder properties such as the cohesive strength, mass flow rate and particle size and shape have a profound impact on filling efficiency. In gravity filling, the escaping of the air from the die is of particular importance, highlighted by the fact that materials with high permeability showed the most favorable filling behavior.

However, gravity filling is employed only under special circumstances, e.g. when filling any but the first layer of a multilayer tablet. As most tablets are single layered, the usual mechanism of die filling is suction filling. When suction filling is employed, the punch enters the filling zone with the punch face being flush with the die opening. The fill cam then guides the downward motion of the punch directly under the powder bed, creating a differential pressure Δp, the magnitude of which is determined mostly by the punch velocity and permeability of the material. Powder is actively transported into the dies by this mechanism, while simultaneously offering the benefit that only little air counteracting powder flow has to escape the die compared with gravitational filling.

The model for gravity filling was expanded towards suction filling (Schomberg et al., 2025). The fit parameters for calculating the critical paddle speed, the rotation-specific fill volume and reference volume flow were both found to correlate well with an estimated Δp based on the lower punch velocity. The study also showed permeability to be a crucial factor, as it determines the extent of Δp and thus, the material's susceptibility to suction filling.

This was hypothesized earlier by Mills and Sinka, who showed that the effectiveness of suction filling increases with decreasing particle size of microcrystalline cellulose (Mills and Sinka, 2013). Several studies with a linear filling device also demonstrated the general superiority of suction filling over gravity filling, as the critical shoe velocities, representing the maximum speed of the feed shoe that still yields completely filled dies, increased significantly depending on the punch velocity (Baserinia and Sinka, 2019; Jackson et al., 2007; Mills and Sinka, 2013; Sinka et al., 2009). Zakhvatayeva et al. showed that a lactose grade of low air permeability yielded substantially lower filling efficiency under conditions of reduced suction filling, whereas materials of higher permeability gave high filling efficiencies under the same conditions (Zakhvatayeva et al., 2019).

Most of the presented studies investigated die filling and the mechanisms involved on custom testing rigs and rotary presses. To the authors' knowledge, no published literature has ever considered the die filling behavior of pharmaceutical powders on a compaction simulator. Therefore, this study investigates the degree of die filling achieved by model excipients on a compaction simulator. For reference, comparative experiments are conducted on a laboratory scale rotary press. Multiple process setups, especially regarding fill cam geometry, are systematically studied by process parameter variation and elucidated with respect to the relevant filling mechanisms. The filling mechanisms are discussed and mechanistically complemented with physics-based calculations of the course of the differential pressure during filling.

## 2 Materials and methods

### 2.1 Materials

Anhydrous dicalcium phosphate (DCP, DI-CAFOS®-A150, Chemische Fabrik Budenheim KG, Budenheim, Germany), microcrystalline cellulose (MCC, VIVAPUR® 102, JRS Pharma GmbH & Co. KG, Rosenberg, Germany) and α-lactose monohydrate (LAC, GranuLac® 200, Meggle GmbH & Co. KG, Wasserburg, Germany) were used as model excipients of excellent, medium and poor flow behavior. DCP and LAC were lubricated with 1% magnesium stearate (MgSt, Ligamed MF-2-V, Peter Greven GmbH & Co. KG, Bad Münstereifel, Germany) by blending in a Turbula® blender (T2F, Willy A. Bachofen AG, Muttenz, Switzerland) for 15min at $49\;\mathrm{mi}n^{-1}$. The comparably long blending time was chosen as to not foster additional lubricant dispersion and changes in flow properties by the feed frame passage during the tableting experiments. As MCC requires no lubrication, none was applied to preserve its inherent flow properties. All material characterization was carried out using the lubricated materials, when applied.

### 2.2 Methods

#### 2.2.1 Material characterization

Particle sizes of the blends were determined by laser diffraction (Mastersizer® 3000) equipped with a dry dispersion unit (AERO S; both Malvern Panalytical Ltd., Worcestershire, UK) at a dispersion pressure of 0.5bar.

A ring shear tester (RST-XS, Dr. Dietmar Schulze GmbH, Wolfenbüttel, Germany) equipped with a 73ml shear cell was used to determine flow properties. Pre-shear stresses of 1, 2, 4 and 8kPa were applied to the powder blends with shear to failure stresses of 20, 50 and 80% (33% for 1kPa). The free flow coefficient $ff_{c}$ is calculated according to Eq. (1) where $\sigma_{1}\;\left[\mathrm{kPa}\right]$ is the consolidation stress and $\sigma_{0}\;\left[\mathrm{kPa}\right]$ the unconfined yield strength.

(1) $$ff_{c}=\frac{\sigma_{1}}{\sigma_{0}}$$

To assess the powders' cohesion under dynamic conditions, the cohesive index was determined with a rotating drum (GranuDrum™, Granutools, Awans, Belgium). The drum was accelerated to 2, 5, 8 and $10\;\mathrm{mi}n^{-1}$ (rotations per minute). At each speed 30 frames were collected with a sampling interval of 500ms and analyzed using the accompanying software (version: 9.23.8.29). For LAC, the glass windows of the cylinder were manually dusted with MgSt to reduce sticking.

Bulk and tapped density ($\rho_{b}\left[\frac{g}{cm^{3}}\right]$ and $\rho_{t}\left[\frac{g}{cm^{3}}\right]$, respectively) were determined according to Ph. Eur. 11.0 2.9.34 with a 100ml graduated cylinder. The tapping was carried out with tapped density analyzer (Erich Tschacher Laboratoriumsbedarf, Bielefeld, Germany). The compressibility index CI was calculated according to Eq. (2).

(2) $$\mathrm{CI}=\frac{\rho_{t}-\rho_{b}}{\rho_{t}}\cdot 100$$

The particle density $\rho_{s}\left[\frac{g}{cm^{3}}\right]$ was determined by helium pycnometry (Ultrapyc 1200e, Quantachrome Instruments, Florida, USA) with a $131.7\;{\mathrm{cm}}^{3}$ sample chamber. The chamber was purged 10 times before the start of a measurement. For each measurement, only the last three of ten cycles were included in the analysis.

With a powder rheometer (MCR 302, Anton Paar, Graz, Austria), the air permeability $K\;\left[m^{-2}\right]$ was determined. 80ml of powder was filled into the powder cell and fluidized for 200s with a volume flow ramp from $5-0.15\;l\cdot \mathrm{mi}n^{-1}$. The volume flow was than raised back to $5\;l\cdot \mathrm{mi}n^{-1}$ over a subsequent 200s. Throughout the fluidization, the powder was continuously stirred with a blade stirrer rotating at $5\;\mathrm{mi}n^{-1}$. Following fluidization, an air-permeable punch was installed that exerted a normal stress of 1kPa on the powder bed. Two different volume flows, 0.4 and $0.6\;l\cdot \mathrm{mi}n^{-1}$, were applied from the bottom of the powder bed. Darcy's law was used to calculate the permeability by relating the applied volume flow to the resulting differential pressure (Darcy, 1856; Muskat, 1937).

All measurements were carried out in triplicate and the mean and standard deviation were calculated.

#### 2.2.2 Tableting experiments

The experimental parameters are listed in Table 1. All possible combinations of machine, fill cam, material, turret and paddle speed were tested. As paddle speeds can only be set in increments of $3\;\mathrm{mi}n^{-1}$ on the compaction simulator, the presented values were chosen to be as close to those on the rotary press as possible.

**Table 1:** Experimental parameters of the tableting experiments.

Machine	Fill cam	Material	Turret speed [min^−1^]	Paddle speed [min^−1^]
Rotary press	conventional	DCP	20, 30, 40, 50, 60	5, 10, 20, 30, 40, 50, 60
		MCC		
		LAC		
	stearate	DCP		
		MCC		
		LAC		
Compaction simulator	conventional	DCP	20, 30, 40, 50, 60	6, 9, 21, 30, 39, 51, 60
		MCC		
		LAC		
	stearate	DCP		
		MCC		
		LAC		

A laboratory scale rotary tablet press (XL100, KORSCH AG, Berlin, Germany) was equipped with 4 sets of round, flat faced EU-D tools with a diameter of 9mm on a mixed turret rotating in counterclockwise direction. A feed frame designed for the operation with an external lubrication unit was installed with a single paddle wheel with twelve spokes of square geometry rotating in counter clockwise direction. The spoke height on the rotary press feed frame is 6mm. The filling and dosing height were kept constant at 16mm and 15.5mm, respectively.

**Fig. 1:**
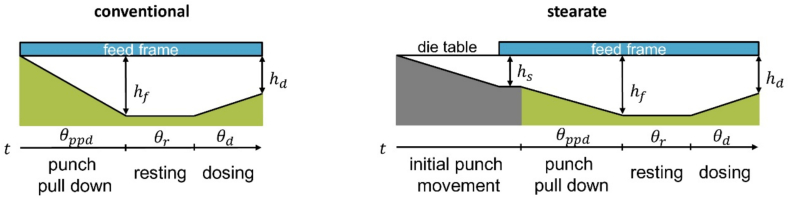
Schematic overview of the investigated fill cams with the filling height $h_{f}$ and dosing height $h_{d}$.

Fig. 1 schematically illustrates the investigated fill cams. The conventional fill cam ensures that the punch is pulled down entirely under the powder bed. The stearate fill cam pulls down the punch to a height of 4.7mm ($h_{s}$) before entering the filling zone, which is necessary if external lubrication is desired on the rotary press. No external lubrication was applied in this study, as this fill cam was employed for its alternative geometry. The remaining filling height is traversed under the powder bed. Both fill cams occupy the same angular sectors θ of the die table for punch pull down, resting and dosing.

The compression distance between the punches was set to an arbitrary value to achieve compression forces adequate to yield sufficiently strong tablets for analysis, without taking into account compressibility or compactibility. For each set of paddle and turret speed, tablets were collected and weighed with an automatic tablet tester (Multicheck 6, Erweka GmbH, Langen, Germany) equipped with an analytical balance (Practum® 124-1S, Sartorius, Göttingen, Germany) (n=20).

The filling yield ϕ, a dimensionless and material-independent measure of the degree of die filling, was calculated from the individual tablet weights according to Eq. (3).

(3) $$\phi =\frac{m_{t}}{\pi *r_{\mathrm{die}}^{2}*h_{d}*\rho_{b}}$$

where $m_{t}\;\left[\mathrm{mg}\right]$ is the tablet weight, $r_{\mathrm{die}}\;\left[\mathrm{mm}\right]$ is the radius of the die, $h_{d}\;\left[\mathrm{mm}\right]$ is the dosage height and $\rho_{b}$ is the bulk density. Here, $\rho_{b}$ is chosen as the reference density, as the actual powder density during the feeder passage and die filling process is unknown and probably subject to changes based on the process parameters. Prior investigations showed, that for free flowing powders the obtained tablet mass is approximately equal to the theoretical tablet mass of a powder volume calculated with the dimensions of the die and $\rho_{b}$, thus yielding ϕ=1.

The compaction simulator (STYL'One Evo, Medelpharm SAS, Beynost, France) was equipped with the same tooling as the rotary press. The standard paddle feed frame provided by the manufacturer was installed with the six-spoked paddle wheel rotating in clockwise direction with a vertically oriented flat face facing the powder. The spoke height of the feed frame on the compaction simulator is 11mm.

The compression profile for the simulation was designed to imitate the geometry of the rotary press using the software Profil'One (Medelpharm SAS, Beynost, France). To accurately reproduce the geometry of the stearate fill cam, the punch was moved to a height of 4.7mm before the feed frame came into contact with the die. Filling and dosing height were set to 16mm and 15.5mm, respectively.

The compaction simulator was operated in force driven mode with a target compression force of 10kN to achieve tablets of sufficient strength for handling and analysis. For each parameter set, tablets were collected and weighed using an analytical balance (Quintix®224 - 1CEU, Sartorius, Göttingen, Germany) (n=10). The filling yield ϕ was calculated according to Eq. (3).

#### 2.2.3 Lower punch dynamics

To evaluate the resemblance of the original filling cams in the simulation, the punch pull down time and velocity were compared over scales. On the rotary press, the linear punch pull down time $t_{\mathrm{ppd}}\;\left[s\right]$ was calculated according to Eq. (4).

(4) $$t_{\mathrm{ppd}}=\frac{m_{t}}{n_{t}\cdot 360^{{}^{\circ}}}$$

where $\theta_{\mathrm{ppd}}\;\left[{}^{\circ}\right]$ is the punch pull down angle.

The linear punch pull down velocity $v_{\mathrm{ppd}}\;\left[\frac{\mathrm{mm}}{s}\right]$ was thus calculated according to Eq. (5).

(5) $$v_{\mathrm{ppd}}=\frac{h_{\mathrm{ppd}}}{t_{\mathrm{ppd}}}$$

with $h_{\mathrm{ppd}}\;\left[\mathrm{mm}\right]$ being the effective punch pull down height under the powder bed.

**Fig. 2:**
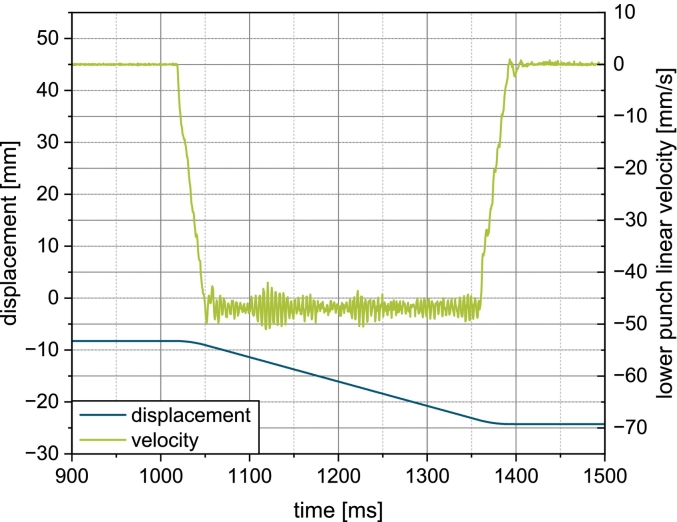
Lower punch displacement and linear velocity for the simulated conventional fill cam a simulated turret speed of 20 min^−1^.

The instrumentation of the compaction simulator provides detailed information about the lower punch displacement and velocity (Fig. 2) gathered at an acquisition frequency of 2000Hz. For each simulated fill cam, n=10 reports were analyzed per simulated turret speed. Each report was processed using a custom python script that determined the punch pull down time as well as the linear punch pull down velocity according to Eq. (5). For both parameters, the mean and standard deviation were calculated.

A second custom python script was utilized to analyse the reported linear velocity of the lower punch. The mean and standard deviation of the stationary velocity was calculated for each report. From this, the inverse-variance weighted mean $\mu_{\text{weighted}}$ and weighted standard deviation $\sigma_{\text{weighted}}$ were calculated according to Eqs. (6), (7) to provide the actual stationary punch pull down velocity for a given turret speed. Additionally, the acceleration and deceleration were determined by linear regression.

(6) $$\mu_{\mathrm{weighted}}=\frac{\sum_{i}\frac{\mu_{i}}{\sigma_{i}^{2}}}{\sum_{i}\frac{1}{\sigma_{i}^{2}}}$$

(7) $$\sigma_{\mathrm{weighted}}=\sqrt{\frac{1}{\sum_{i}\frac{1}{\sigma_{i}^{2}}}}$$

#### 2.2.4 Physics-based calculation of the differential pressure during punch pull down

To investigate the material-dependent discrepancies in suction filling of the geometries and machine scales, a numerical, physics-based calculation of differential pressure inside the die was derived using an ordinary differential equation (ODE). The equation is based on the works of Schomberg et al. who considered the stationary case of a constant punch pull down velocity for model development (Schomberg et al., 2025). This model is able to provide an estimate for the conditions on rotary presses with fill cams of conventional geometry, as a constant punch pull down velocity can be assumed due to the guided punch movement and there is no air volume inside the die when it enters under the powder bed in the feed frame. For a stearate fill cam of the geometry used in this study, the initial air volume acts as a buffer against the build-up of a differential pressure across the powder bed. Furthermore, the acceleration and deceleration of the punch on the compaction simulator cannot be accounted for in the model of Schomberg et al. as it only reflects stationary conditions.

To enable the calculation via an ODE, the model rests on the following assumptions:

1. The air is considered to be an ideal gas
2. The powder bed height is assumed to be constant across the cross-sectional area of the die
3. Material movement, including material entering the die, is neglected
4. Airflow through the gaps between lower punch and die wall is neglected
5. The process is assumed to be isothermal

While this idealized model equation will not predict the true conditions inside the die, it provides valuable insight into the complex interrelationship of material and process while requiring only little computational resources. A coupled CFD/DEM simulation to model suction filling on linear filling devices was proposed by Wu et al. and showed that, despite particles entering the die immediately, a relevant differential pressure builds up (Wu and Guo, 2012). A similar in silico study reflecting the conditions and geometries of the feed frames of a compaction simulator and rotary press should be conducted in the future.

Model development starts with the ideal gas law, differentiating pressure $p\;\left[\mathrm{Pa}\right]$, volume $V\;\left[m^{3}\right]$ and amount of substance of air $n\;\left[\mathrm{mol}\right]$ with respect to time.

(8) $$\frac{d}{\mathrm{dt}}\left(p\cdot V\right)=\mathrm{RT}\cdot \frac{\mathrm{dn}}{\mathrm{dt}}$$

(9) $$V\left(t\right)\cdot \frac{\mathrm{dp}}{\mathrm{dt}}+p\left(t\right)\cdot \frac{\mathrm{dV}}{\mathrm{dt}}=\mathrm{RT}\cdot \frac{\mathrm{dn}}{\mathrm{dt}}$$

$R\left[\frac{J}{\mathrm{mol}\cdot K}\right]$ is the universal gas constant and $T\;\left[K\right]$ the absolute temperature. The function $V\left(t\right)$ describes how the volume expands over time and comprises the initial volume $V_{0}$ and the incremental changes in volume as the punch moves downward. The change in volume is given as the product of the height of the expanding cavity $h\left(t\right)\;\left[m\right]$ and the cross sectional area of the die $A_{\mathrm{die}}\;\left[m^{2}\right]$:

(10) $$V\left(t\right)=V_{0}+A_{\mathrm{die}}\cdot h\left(t\right)$$

Consequently, $\frac{\mathrm{dV}}{\mathrm{dt}}$ can be expressed as follows, where $v\left(t\right)\;\left[\frac{m}{s}\right]$ is the punch velocity:

(11) $$\frac{\mathrm{dV}}{\mathrm{dt}}=A_{\mathrm{die}}\cdot v\left(t\right)$$

The incremental change in amount of substance of air $\frac{\mathrm{dn}}{\mathrm{dt}}$ can be expressed by the following expression, where $Q_{\mathrm{air}}\;\left[\frac{m^{3}}{s}\right]$ is the air volume flow, $\rho_{\mathrm{air}}\;\left[\frac{\mathrm{kg}}{m^{3}}\right]$ is the density of air and $M_{\mathrm{air}}\;\left[\frac{\mathrm{kg}}{\mathrm{mol}}\right]$ is the average molar mass of air:

(12) $$\frac{\mathrm{dn}}{\mathrm{dt}}=Q_{\mathrm{air}}\cdot \frac{\rho_{\mathrm{air}}}{M_{\mathrm{air}}}$$

To incorporate the powder bed into the equation, $Q_{\mathrm{air}}$ is substituted with Darcy's law, where K is the material's air permeability at a normal stress of 1kPa (see Section 2.2.1), $\eta_{\mathrm{air}}\;\left[\mathrm{Pa}\cdot s\right]$ is the dynamic viscosity of air and $L\;\left[m\right]$ is the powder bed height. The differential pressure across the powder bed is given by the ambient pressure $p_{\mathrm{atm}}$ and $p\left(t\right)$ inside the die.

(13) $$Q_{\mathrm{air}}=\frac{K\cdot A_{\mathrm{die}}}{\eta_{\mathrm{air}}*L}\cdot \left(p_{\mathrm{atm}}-p\left(t\right)\right)$$

(14) $$\frac{\mathrm{dn}}{\mathrm{dt}}=\frac{K\cdot A_{\mathrm{die}}\cdot \rho_{\mathrm{air}}}{\eta_{\mathrm{air}}\cdot L\cdot M_{\mathrm{air}}}\cdot \left(p_{\mathrm{atm}}-p\left(t\right)\right)$$

Substituting these expressions into Eq. (9) gives the ODE that can be solved for $\frac{\mathrm{dp}}{\mathrm{dt}}$:

(15) $$\left(V_{0}+A_{\mathrm{die}}\cdot h\left(t\right)\right)\cdot \frac{\mathrm{dp}}{\mathrm{dt}}+p\left(t\right)\cdot A_{\mathrm{die}}\cdot v\left(t\right)=\mathrm{RT}\cdot \frac{K\cdot A_{\mathrm{die}}\cdot \rho_{\mathrm{air}}}{\eta_{\mathrm{air}}\cdot L\cdot M_{\mathrm{air}}}\cdot \left(p_{\mathrm{atm}}-p\left(t\right)\right)$$

(16) $$\frac{\mathrm{dp}}{\mathrm{dt}}=\frac{\mathrm{RT}\cdot \frac{K\cdot A_{\mathrm{die}}\cdot \rho_{\mathrm{air}}}{\eta_{\mathrm{air}}\cdot L\cdot M_{\mathrm{air}}}\cdot \left(p_{\mathrm{atm}}-p\left(t\right)\right)-p\left(t\right)\cdot A_{\mathrm{die}}\cdot v\left(t\right)}{V_{0}+A_{\mathrm{die}}\cdot h\left(t\right)}$$

The powder bed height L is chosen based on the spoke height of the paddle wheels following the approach of Schomberg et al. As in this case $V_{0}$ can be expressed as $V_{0}=A_{\mathrm{die}}\cdot h_{0}$ with $h_{0}\;\left[m\right]$ being the initial punch position, dividing by $A_{\mathrm{die}}$ and introduction of a constant $c\;\left[\frac{m}{s}\right]$ gives the final model equation:

(17) $$c=\mathrm{RT}\cdot \frac{K\cdot \rho_{\mathrm{air}}}{\eta_{\mathrm{air}}\cdot L\cdot M_{\mathrm{air}}}$$

(18) $$\frac{\mathrm{dp}}{\mathrm{dt}}=\frac{c\cdot \left(p_{\mathrm{atm}}-p\left(t\right)\right)-p\left(t\right)\cdot v\left(t\right)}{h_{0}+h\left(t\right)}$$

$h\left(t\right)$ is calculated from $v\left(t\right)$ at any point on the curve of time $t\;\left[s\right]$. $v\left(t\right)$ is assumed to be constant on the rotary press due to the guided movement by the turret rotor on a linear slope. On the compaction simulator, $v\left(t\right)$ is determined by the acceleration, stationary velocity and deceleration of the punches, which depends on the selected turret speed. Therefore, for each phase of the punch movement, a second degree polynomial regression function $f\left(n_{t}\right)$ was derived from the reports provided by the compaction simulator. The regression parameters as well as all constants (see Supplementary Material Table 1 and Eqs. (1), (2), (3), (4), (5), (6), (7), (8)) were transferred into a python script, that numerically solved the above equation to yield the differential pressure inside the die for a given turret speed and permeability. 300 points of the curve spanning the filling time obtained by Eq. (4) were calculated using an implicit Runge-Kutta method of Radau IIA family order 5 as provided in SciPy 1.15.2 (Hairer and Wanner, 1996; Virtanen et al., 2020). Relative and absolute tolerance were set to $10^{-6}$ and $10^{-8}$, respectively, yielding a relative error in the maximum pressure of ≤0.1% in all cases. The reference solution was calculated with relative and absolute tolerances of $10^{-12}$ and $10^{-14}$, respectively.

To avoid division by zero in case of the conventional fill cam where $h_{0}=0$, calculation starts at $t=10^{-6}$. The same is applied to the calculation of the stearate fill cam to keep calculations uniform.

## 3 Results and discussion

### 3.1 Material characterization

**Fig. 3:**
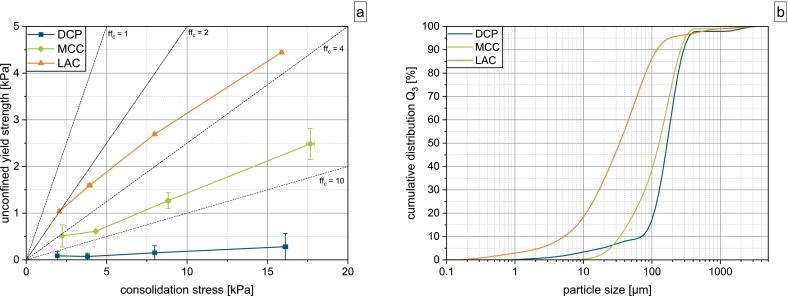
a) Flow function and b) cumulative particle size distribution of DCP, MCC and LAC.

According to the classification of Jenike, with $ff_{c}$ in the range of 2−4, LAC can be classified as a cohesive material, while MCC is considered easy flowing with an $ff_{c}$ value in the range of 4−10 (Jenike, 1976) (Fig. 3). With $ff_{c}\;\gg \;10$, DCP is considered free flowing. The results of the powder characterization are given in Table 2.

**Table 2:** Powder characteristics of investigated materials.

Material	$x_{10}$[μm]	$x_{50}$[μm]	$x_{90}$[μm]	$\rho_{b}$[g/cm^3^]	$\rho_{t}$[g/cm^3^]	$\rho_{s}$[g/cm^3^]	CI[%]	K[m^2^]
DCP	70.2 ± 4.0	167.0 ± 0.0	292.3 ± 10.7	0.738 ± 0.009	0.870 ± 0.008	2.838 ± 0.008	15.20 ± 0.3	5.20E-12 ± 1.18 E-14
MCC	34.1 ± 0.5	127.0 ± 2.6	263.3 ± 11.6	0.355 ± 0.006	0.455 ± 0.004	1.595 ± 0.001	22.0 ± 0.9	4.62E-12 ± 3.18E-13
LAC	5.3 ± 0.1	33.7 ± 0.4	118.5 ± 4.4	0.528 ± 0.004	0.784 ± 0.006	1.548 ± 0.005	32.6 ± 0.9	5.61E-13 ± 1.08E-13

### 3.2 Die filling experiments

#### 3.2.1 Free flowing and dense powder

**Fig. 4:**
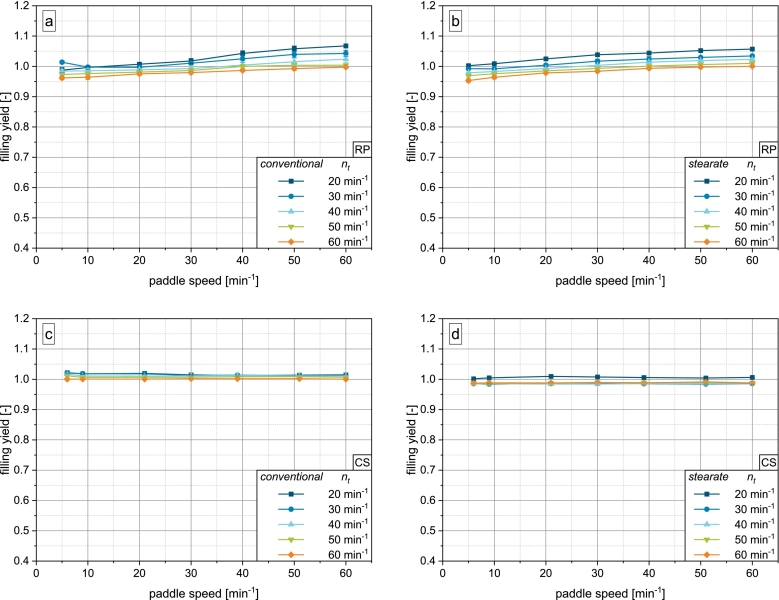
Results of die filling experiments for DCP: a) conventional fill cam on rotary press, b) stearate fill cam on rotary press, c) conventional fill cam on compaction simulator, d) stearate fill cam on compaction simulator.

The study shows that regardless of machine and fill cam geometry, the free flowing and dense model powder DCP achieves highly filled dies at all combinations of turret and paddle speed (Fig. 4). Weight variation is consistently below 2% (see Supplementary Material Fig. S.1). On the rotary press, two general trends can be identified: The higher the paddle speed and the lower the turret speed, the higher the filling yield. This is in accordance with the literature (Schomberg et al., 2023a; Schomberg et al., 2020; Tang et al., 2020) and can be explained by the availability of powder to the die. At low turret speeds, the filling time during which the dies move under the opening of the feed frame is prolonged, giving the powder more time to flow into the dies. At high paddle speeds, the powder transport to the filling zone of the feed frame is increased.

On the compaction simulator, the filling yield of DCP does not depend on paddle speed, which can be explained by the machine geometry and consequent discrepancies in acting filling mechanisms. As the compaction simulator has a stationary compression station, the punches are limited to vertical movement, while on the rotary press the tools' movement comprises a vertical and circumferential component. The pressure differential across the powder bed and a resulting airflow created by the downward motion of the punch is therefore concentrated to a much smaller space directly above the die on the compaction simulator. In contrast, the suction effect it is spread out over the opening of the feed frame along the track of the die on the rotary press, rendering it less efficient. In addition, due to paddle wheel on the compaction simulator having only half the number of spokes, the number of paddle passes (paddle-die-contacts) is reduced with respect to the rotary press. This is further amplified by the fact, that the die is stationary on the compaction simulator with the paddle wheel simply passing over it. On the rotary press, under the presented operating conditions, where both die table and paddle wheel rotate in counterclockwise direction, powder is passed towards the die which itself is moving towards the powder, greatly aiding the active powder feeding. In addition, the contact area of die and powder is limited to the cross-sectional area of the die on the compaction simulator due to the stationary nature of the compression station. On the rotary press however, as the die passes the opening of the feed frame, the cumulative contact area with the powder is much larger. For these reasons, the paddle rotation is able to provide less powder to the die in the same time on the compaction simulator. Together, the increased suction effect and reduced powder transport by the paddle wheel on the compaction simulator serve to explain the discrepancy observed in Fig. 4.

The data shows that the mechanism of suction filling determines the filling yield on the compaction simulator to a much higher degree than on the rotary press, where the powder availability, which is determined by both paddle speed and filling time, is the determining factor. As DCP is a material of excellent flowability and high particle density, its filling behavior is also governed by gravity, which serves as an explanation as to why at a turret speed of $20\;\mathrm{mi}n^{-1}$ with the lowest intensity of suction filling and longest filling time, completely filled dies are obtained independent of paddle speed. The air present in the partially opened die due to the geometry of the stearate fill cam shows no considerable effect on the filling yield of DCP. This might also be due to the high particle density, which was shown to affect the sensitivity towards counteracting air flow (Guo et al., 2009).

Overfilling of the dies represented by ϕ>1 is observed in all setups and can be attributed to powder densification, where the density during dosing out is $\rho >\rho_{b}$. Powder can be compressed both by the stress applied by the paddle wheel as well as by the upward movement of the punch during dosing out. If the die was completely filled initially, the material inside the die may be compressed against the powder bed above. Schomberg et al. showed that the degree of overfilling increases with paddle speed and overfill ratio (overfill/filling height) and decreases with turret speed (Schomberg et al., 2020). As in this study, the overfill ratio is very small, only the slight overfilling on the compaction simulator can be explained this way. On the rotary press, powder is likely additionally compressed by the paddle rotation. As the number of spokes on the rotary press is twice that of the compaction simulator's feed frame, the stress intensity is expected to be greater as by the concept of the number of paddle passes introduced by Mendez et al. (Mendez et al., 2010). However, to verify this hypothesis, investigations into residence time distributions in compaction simulator feed frames are necessary. The lower degree of overfilling on the compaction simulator can also be explained by the increased suction effect experienced by the powder whereby it may be aerated, leading to a decreased in-die density. Additionally, the higher degree of overfilling is likely also a result of stronger vibrations on the rotary press than on the compaction simulator, especially at high turret speeds.

#### 3.2.2 Easily flowing and highly compressible powder

**Fig. 5:**
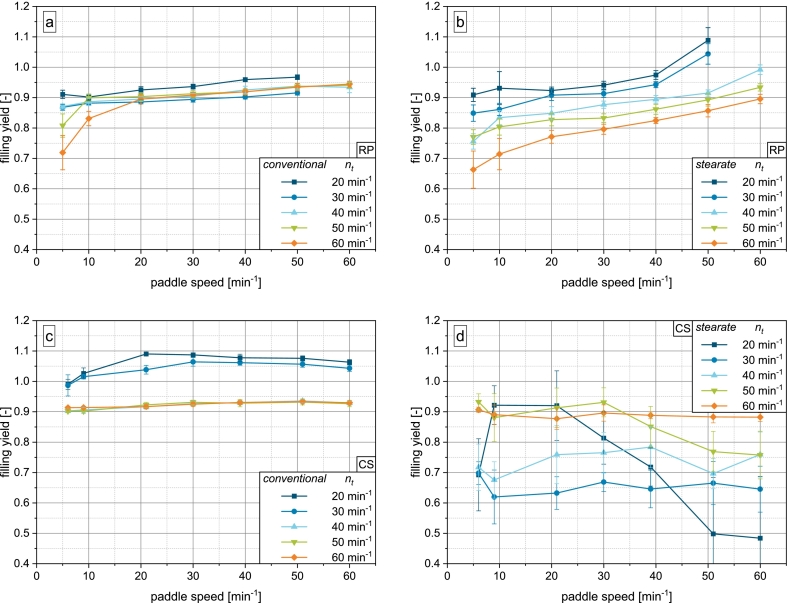
Results of die filling experiments for MCC: a) conventional fill cam on rotary press, b) stearate fill cam on rotary press, c) conventional fill cam on compaction simulator, d) stearate fill cam on compaction simulator.

For MCC as an easily flowing and highly compressible powder, a clear impact of machine scale and fill cam geometry emerges (Fig. 5).

On the rotary press, the same trends regarding turret and paddle speed hold true. With the conventional fill cam, paddle speeds from $20\;\mathrm{mi}n^{-1}$ on yield a comparable degree of die filling with little dependence on turret speed. Only at $20\;\mathrm{mi}n^{-1}$ turret speed, slightly higher filling yields are observed, which may be caused by the prolonged filling time. Paddle speeds of 5 and $10\;\mathrm{mi}n^{-1}$ are not able to provide enough powder to the dies at high turret speeds, as the low flowability of MCC acts as the limiting factor for powder transport under these conditions (Schomberg et al., 2020). For MCC, a paddle speed of $60\;\mathrm{mi}n^{-1}$ could not be investigated at turret speeds of 20 and $30\;\mathrm{mi}n^{-1}$, as the low powder demand by the dies led to increased powder compression inside the feed frame, risking damage to the machine.

With the stearate fill cam, a clear discrimination of filling yield by turret speed is visible throughout the process. Especially at high turret speeds, the obtained filling yields are noticeably lower than with the conventional fill cam. Only at high paddle speeds, degrees of filling with the stearate cam approach those of the conventional cam.

The impact of fill cam geometry on the filling behavior of MCC on the rotary press is explained by the underlying filling mechanisms. Since both paddle rotation frequencies and the gravitational forces are the same in both setups, a reduction in the extent of suction filling is identified as the cause of the reduced filling efficiency of the stearate fill cam. As the punch enters the filling zone partially pulled down, the stearate fill cam can be classified as a blend of suction and gravity filling. The implications of this are discussed further in section 3.3. In addition, the escaping of the air inside the partially opened die introduced by the stearate fill cam will counteract powder flow, especially as MCC is of lower density and lower permeability than DCP. This is a well known issue in the case of pure gravity filling and will thus also occur with the stearate fill cam, albeit to a lesser degree (Guo et al., 2009; Wu et al., 2003).

Interestingly, completely filled dies are never achieved with the conventional fill cam, while the stearate fill cam even yields overfilled dies at low turret and high paddle speeds. This can also be explained by the different degrees of suction filling. The faster punch pull down velocity on the conventional fill cam creates a greater airflow through the powder bed. This may lead to the inflowing air aerating the powder, which is filled into the die. Accordingly, the apparent in-die density of the filled material may be lower than the bulk density, resulting in ϕ<1 even though the die is completely filled by volume without macroscopic voids. During dosing out, the powder is mostly deaerated inside the die instead of compressing it perceptibly against the powder bed above.

On the stearate fill cam however, the reduced airflow by the lower punch pull down velocity is not able to aerate the powder in a similar manner. Consequently, the apparent in-die density is closer to or even greater than the bulk density of the material, especially at high paddle rotation frequencies, where the highly compressible material is densified in the feed frame. Similarly to DCP, this is not observed on the compaction simulator, possibly due to the reduced number of paddle passes and higher aeration during suction filling.

MCC shows substantially different filling behavior on the compaction simulator (Fig. 5c – d). With the simulated conventional fill cam, the filling yields obtained at simulated turret speeds of $40-60\;\mathrm{mi}n^{-1}$ are similar to the rotary press for paddle speeds from $20\;\mathrm{mi}n^{-1}$ on (cf. Fig. 5a). The limitation in filling yield at low paddle speeds was not reproduced on the compaction simulator. At $20-30\;\mathrm{mi}n^{-1}$ turret speed, overfilling occurred unexpectedly. Tablet weight variation is consistently low for all investigations applying the conventional fill cam simulation (see Supplementary Material Fig. S.2).

In contrast, the simulated stearate fill cam gives a very high tablet weight variation and vastly differing filling yields. With regard to paddle speed, no clear trends emerge. Overall, the filling yield increases with turret speed in full contradiction to rotary press results, showing that suction filling is the dominating filling mechanism on the compaction simulator in this, rendering the effect of turret speed on filling time less crucial. Consequently, the reduction of the punch pull down velocity and suction pressure due to the stearate fill cam simulation has a stronger impact on the filling yield of MCC on the compaction simulator.

**Fig. 6:**
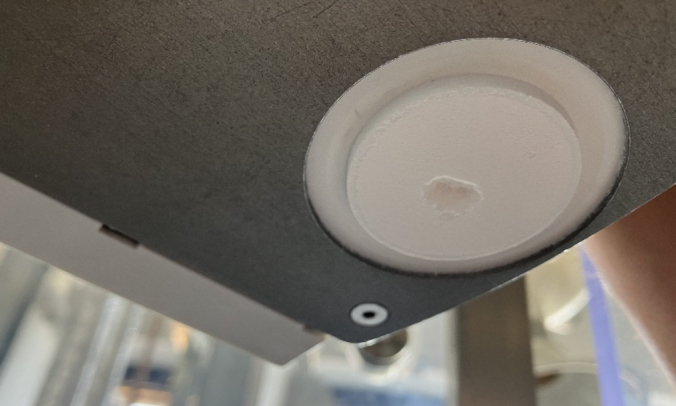
MCC bridging in the powder outlet of the paddle feed frame of the compaction simulator after applying the sequence of 5 –60 min^−1^ paddle speed at 20 min^−1^ turret speed.

Additionally, the ineffectiveness of the paddle speed variation to influence filling of MCC on the compaction simulator pronounces the differences in achieved powder availability to the die. This is particularly supported by the peculiar results for a simulated turret speed of $20\;\mathrm{mi}n^{-1}$. The initial increase and subsequent decrease in filling yield can be attributed to increased powder densification inside the outlet of the feed frame (i.e. below the paddle wheel and above the die) with increasing paddle speed. This results in bridging (Fig. 6) due to the high compressibility (Table 2) and limited flowability (Fig. 3a) of MCC. As no similar effect is seen with the conventional profile, its higher suction effect by increased punch pull down velocity is likely able to break any bridges inside the powder outlet, even at lower simulated turret speeds.

#### 3.2.3 Poorly flowing and cohesive powder

**Fig. 7:**
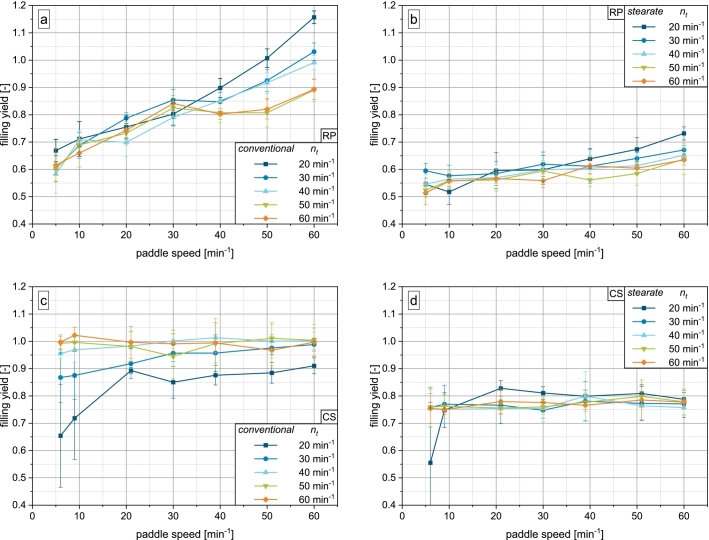
Results of die filling experiments for LAC: a) conventional fill cam on rotary press, b) stearate fill cam on rotary press, c) conventional fill cam on compaction simulator, d) stearate fill cam on compaction simulator.

Similarly to MCC, the poorly flowing and cohesive LAC shows a strong dependence of the filling yield on the machine scale and fill cam geometry (Fig. 7), which again can be attributed to the impact of constructional aspects on suction filling, as discussed above.

On the rotary press equipped with the conventional fill cam, the filling yield increases approximately linearly with the paddle speed independent of turret speed up to a paddle speed of $30\;\mathrm{mi}n^{-1}$. This indicates that powder availability to the filling zone and thus the flowability of the material is the limiting factor of filling yield at these process parameters. Even though the filling time is prolonged at low turret speeds, the little material that is provided by the low paddle speeds does not lead to increased filling yields. When the paddle speed exceeds $40\;\mathrm{mi}n^{-1}$, the powder demand by the dies takes over as the limiting factor and a clear discrimination by turret speed and thus filling time emerges.

In contrast, the filling yield on the stearate fill cam on the RP is entirely limited by the availability of powder to the dies and thus, no discrimination by turret speed emerges. The shift of the curves to lower filling yields and reduction in slope can be attributed to a reduction in the degree of suction filling, similar to the observation made by Zakhvatayeva et al. (Zakhvatayeva et al., 2019). This amplifies the limitation by powder availability observed with the conventional fill cam.

Fig. 7c – d show the filling behavior of LAC on the compaction simulator, which again heavily contrasts the results obtained on the rotary press. Simulating the conventional fill cam, the filling yield increases with turret speed, in contrast to the rotary press, yielding completely filled dies from $40\;\mathrm{mi}n^{-1}$. This again pronounces the dominance of suction filling on the compaction simulator. No relevant overfilling is observed. Furthermore, a dependence of filling yield on paddle speed, which is characteristic for the rotary press, can be observed at turret speeds of $20-40\;\min^{-1}$.

In contrast to MCC, suction filling is not able to fill the dies entirely on its own. As LAC shows very poor flowability and high cohesiveness, powder flow is likely not initiated across the entire powder bed, but only localized above the die. With increasing turret speed, the suction effect is able to break of an increasing portion of powder that enters the die. On the rotary press however, the reduced and spread out suction effect is not strong enough to efficiently supply enough powder for complete die filling. Instead, the paddle rotation is necessary to facilitate sufficient powder flow into the dies. Although too high for industrial application purposes, both machines give similar tablet weight variations for both fill cams (see Supplementary Material Fig. S.3).

The results for the simulated stearate fill cam can be explained similarly to that of the rotary press. As the suction pressure is reduced with this configuration, the cohesive powder bed cannot be loosened to the same extent as with the conventional fill cam. Consequently, the maximum filling yield is limited. In contrast to the rotary press, an increase in paddle rotation frequency in the observed range is not able to increase the filling yield. This may be caused by the geometric differences of the feed frames of the rotary press and compaction simulator, especially the area (smaller on compaction simulator) and height (higher on compaction simulator) of the powder-filled opening between paddle and die table. In the future, a deeper investigation into the impact of paddle speed on the compaction simulator is necessary, e.g., by matching the tip speed to the conditions on the rotary press. Additionally, constructive adaptions, e.g., the increase of the opening area, will be of interest.

### 3.3 Mechanistic discussion

#### 3.3.1 Linear punch velocity

The compaction simulator was, independent of fill cam geometry, able to reproduce the filling behavior of DCP, an excellently flowing and dense material. The results for both MCC and LAC, materials of only limited flowability, differed greatly over scales and fill cam geometries, which all can be traced back to discrepancies in acting powder supply and filling mechanisms, particularly suction filling.

To better understand the discrepancies in suction filling over scales and fill cam geometry, the lower punch movement was investigated.

**Fig. 8:**
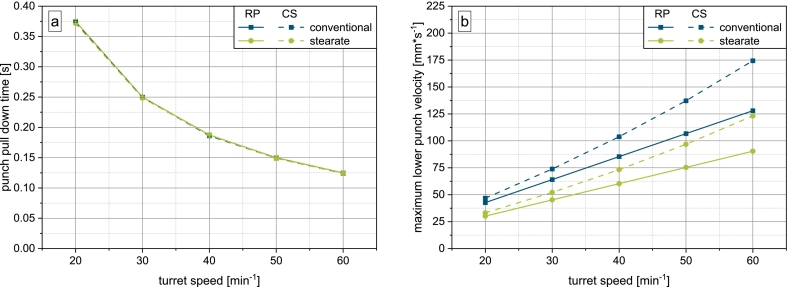
a) Lower punch pull down time and b) maximum velocity for both fill cams on the compaction simulator and rotary press.

The effective punch pull down height under the powder bed of the stearate fill cam is reduced by 4.7mm due to the preliminary punch pull down to enable external lubrication. Since punch pull down under the powder bed occupies the same angular sector on both fill cams, the filling time is the same and thus, the linear punch pull down velocity is reduced for the stearate fill cam (Fig. 8b). Analysis of the punch displacement and velocity on the compaction simulator showed, that while the punch pull down time matches the rotary press (Fig. 8a), the maximum stationary velocity reached during punch pull down is much higher than on the rotary press (Fig. 8b). This discrepancy in the dynamic conditions adds to the differences in the punch movement and paddle feeder geometry on the machines as described in section 3.2.1. In sum, the higher punch velocities and more concentrated airflow serve as an explanation for the increased suction effect on the compaction simulator.

The discrepancy in punch velocity is a consequence of the mechanics of the compaction simulator. On the rotary press, the punch is guided at a constant velocity by the turret, requiring no additional acceleration and deceleration. As the punches are stationary on the compaction simulator, when filling is initiated, the punch is accelerated to a certain stationary velocity, following a deceleration phase to precisely reach the target filling height. The average velocity over the entire punch pull down, calculated from the data of the instrumentation of the compaction simulator, was found to match the linear punch pull down velocity calculated according to Eq. (5).

This however does not take into account two very important issues. Firstly, the acceleration of the punch critically determines how fast a differential pressure builds up across the powder bed. Secondly, the deceleration at the end of the downward motion likely does not contribute much to the die filling process, as most of the powder flow will occur in the beginning of the die filling process during punch acceleration and punch movement at constant velocity.

Furthermore, punch velocity only partially explains the observed behavior as the buffering effect of the initial air pocket that is introduced by the stearate fill cam is not taken into account. For a more fundamental investigation of the impact of material and machine and fill cam geometry, the exerted differential pressure has to be estimated.

#### 3.3.2 Physics-based calculation of differential pressure

**Fig. 9:**
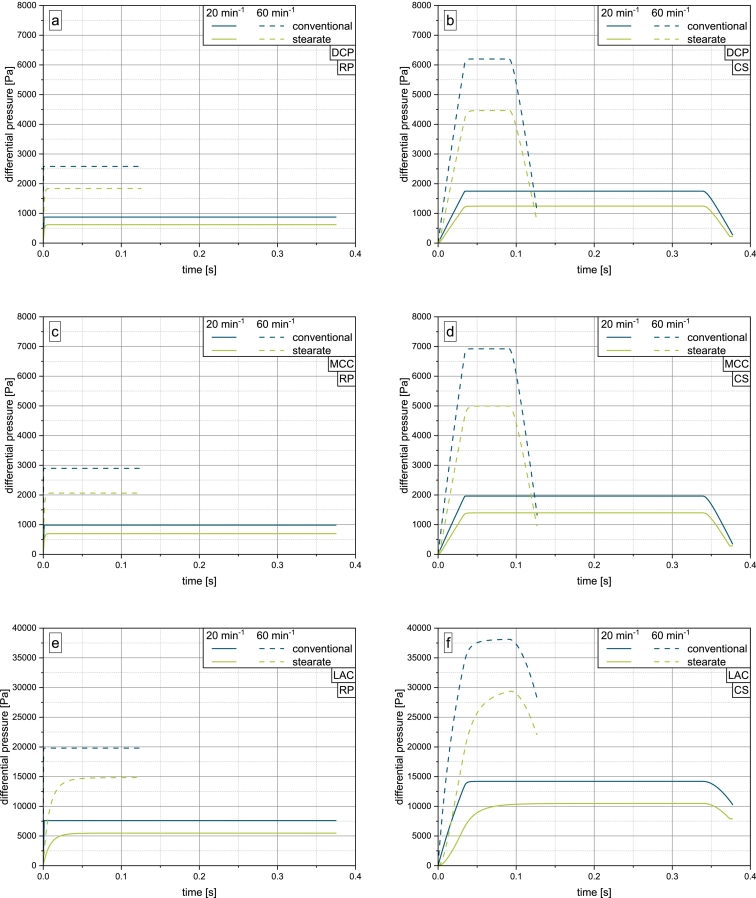
Results of numerical calculation of differential pressure on the rotary press and compaction simulator for DCP (a, b), MCC (c, d) and LAC (e, f) at turret speeds of 20 and 60 min^−1^ (for clear readability, e) and f) are adjusted in scale).

Fig. 9 shows the results of the numerical calculation of the evolving suction pressure according to Eq. (18) for the investigated materials on the rotary press (a, c, e) and compaction simulator (b, d, f). It is readily apparent, that for the rotary press the rotor-guided motion of the punches leads to an instantaneous buildup of a stationary Δp across the powder bed in case of the conventional fill cam. As expected, the calculations show that the initial air pocket in the stearate fill cam, represented by the position of the punch upon entry into the filling zone of the feed frame $h_{0}$, delays the buildup of a stationary Δp. This corresponds well to the experimental results, which indicated a substantially lower degree of suction filling on the stearate fill cam (cf. Figs. 5a, b and 7a, b).

The extent of this effect depends strongly on the material's permeability, as is apparent when comparing Fig. 9c and Fig. 9e. LAC, which exhibits a permeability about 10 times lower than that of MCC (see Table 2), shows a much longer buildup of differential pressure to an also multiple pressure level. Airflow through the powder bed is more limited, delaying the formation of an equilibrium state. The lower stationary Δp for the stearate fill cam is a result of the decreased punch velocity during punch pull down by reduction of the effective punch pull down height. When the travel distance of the punch was set to 16mm, similarly to the conventional fill cam, the calculation yielded the same stationary Δp for both fill cams (data not shown).

On the compaction simulator, where the punches are accelerated and decelerated, the buildup of Δp is not instantaneous, instead following the shape of the lower punch velocity profile (see Fig. 2), whereby the exact shape of the curve is additionally determined by the material's permeability. Again, a slower buildup and lower stationary Δp are observed for the stearate fill cam, matching the expectations. The obtained stationary Δp on the compaction simulator are much higher and can be attributed to the increased punch velocities that result from the machine control. This enforces the particular importance of suction filling as the dominant filling mechanism on the compaction simulator.

Powders with limited flowability and high cohesiveness tend to show bridging behavior over outlets when stored in confined spaces (Jenike, 1976). Even though the residence time in tablet press feed frames is much shorter than under storage conditions, especially with LAC, bridging can be expected to some extent, as the die diameter of 9mm is very small when compared to powder outlets in industrial scale silo storage. Furthermore, in case of the compaction simulators feed frame, the space between the spokes and die is sensitive to bridging due to compression of the material by the paddle rotation (cf. Fig. 5a and Fig. 6). The fact that the powder is not continuously agitated by the paddle rotation on the compaction simulator further increases bridging propensity. On the rotary press, the continuous stirring in the feed frame prevents bridging.

**Fig. 10:**
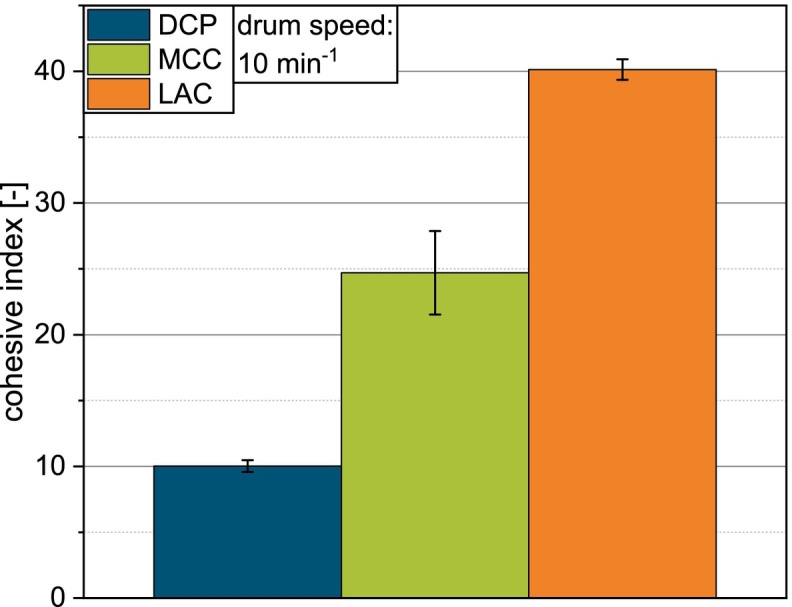
Cohesive index of DCP, MCC and LAC at a drum speed of 10 min^−1^.

If bridges form, they need to be broken by a sufficiently strong force that is transmitted either by the paddle rotation or by differential pressure due to suction filling. On the compaction simulator, the latter seems to be more effective, as indicated by the results of the die filling experiments for MCC (see Fig. 5c and d). As the breaking of bridges is the first step necessary to facilitate powder flow, the rate of increase of Δp is of particular importance. The calculation's results correspond well to the experimental data in this regard, as a generally slower increase of Δp on the stearate fill cam matches deficits in filling efficiency with this geometry (see Fig. 5d). As the slope of $\frac{\Delta p}{t}$ increases with turret speed, bridges are more easily broken, indicated by an increase in filling yield with turret speed. However, this is only observed for MCC. For LAC, the experimental data suggests that indifferent of the investigated turret speed and thus suction pressure, only a limited amount of material directly above the die is set in motion by the resulting airflow. This hypothesis is supported further by the high cohesive index of LAC (see Fig. 10).

The calculation for both machines yields the highest Δp for LAC, which is about 7.5 times higher than for DCP and MCC, which yield similar Δp, corresponding to the materials' permeabilities, which is the lowest for LAC and similar for MCC and DCP. This highlights the dependency of LAC on suction filling to achieve suitable die filling (see Fig. 7). However, the similar results for DCP and MCC suggest both materials to be about equally dependent on suction filling, not matching the experimental data (see Fig. 4 and Fig. 5).

As the model equation only takes the permeability as a powder characteristic, other important material properties that are crucial for sufficient die filling such as the flowability and cohesion are not considered (Anand et al., 2009; Baserinia et al., 2016; Mehrotra et al., 2009; Tang et al., 2020; Zakhvatayeva et al., 2019). Additionally, the very different feed frame geometry of the machines is not considered. It must be taken into account that the flow of air and resulting Δp is more spread out through the larger powder bed in the more voluminous rotary press feed frame. This is due to the movement of the die table under the rotary press feed frame. On the contrary, the die rests steadily under the feed frame with its tight geometry during filling on the compaction simulator.

These findings motivate an extension of the presented model for die filling on rotary presses to make it also fit for the altered conditions on a compaction simulator. However, the current model offers qualitative insight into the mechanism of suction filling, especially with regard to fill cam geometry. CFD/DEM simulations of the entire filling system, comprising feed frame, die and moving punch, may provide more in-depth understanding of the relevant filling mechanisms and their interrelationship with material properties (Wu and Guo, 2012), however dependent on their precision and calibration.

### 3.4 Evaluation of the predictive potential of compaction simulation with respect to die filling

3.2, 3.3 show the shortcomings of the compaction simulator in the physical simulation of die filling on the investigated rotary press. Only for DCP, the material with the most favorable properties for die filling, the trials on the compaction simulator correctly estimated the complete die filling achieved on the rotary press (see Fig. 4). In the case of MCC, the compaction simulator was able to reflect the conditions on the rotary press only for turret speeds $>30\;\mathrm{mi}n^{-1}$ at paddle speeds $>10\;\mathrm{mi}n^{-1}$ (see Fig. 5). For LAC, the compaction simulator systematically overestimated the achievable filling yields on the rotary press (see Fig. 7). In both cases, the higher suction pressure exerted on the powder by the compaction simulator was identified as the main reason for the observed deviations (see Fig. 9). For MCC, the suction pressure masks the inability of low paddle speeds to foster complete die filling as too little material is transported in the short filling time. For LAC, its high cohesiveness and low permeability make it particularly susceptible to the exerted suction pressure, adding to the challenging flowability that inhibits paddle-induced flow into the dies. This leads to the conclusion, that for potentially challenging formulations, which would be of utmost interest for small-scale evaluation, the compaction simulator is not able to produce a meaningful estimate of the filling yield for the investigated rotary press. To address this, the characteristic dynamic and geometric aspects of the compaction simulator have to be compensated. The overestimation of the acting suction pressure on the compaction simulator should be addressed in future studies by systematically varying the lower punch velocity, reducing the exerted suction pressure. Furthermore, alternate feeder geometries should be investigated that increase the impact of paddle rotation on the compaction simulator, e.g. by getting the die table and paddle wheel in closer contact. Both dynamic and geometric factors need to be incorporated into a model for transferring results from the compaction simulator towards rotary presses.

## 4 Conclusion

In this study, model materials of different flowability were processed on a compaction simulator and rotary tablet press under varying process conditions to compare the filling behavior over scales.

It was shown that the filling yield is highly dependent on material flowability on both machines. In addition, materials differed in the mechanisms that drove their filling into the dies. Freely flowing and highly dense DCP was shown to be robust to conditions of reduced suction filling, while MCC and LAC showed a strong dependence on the intensity of suction filling.

Consequently, materials were affected differently over scales, as the acting filling mechanisms depend on machine geometry. On the compaction simulator, the increased lower punch velocities and purely vertical movement generally created a higher and more concentrated differential pressure across the powder bed, resulting in stronger suction filling. The paddle rotation showed no pronounced effect on filling yield. On the rotary press, the suction effect spread out over a larger area and was reduced by the decreased lower punch velocities as well as the combined vertical and circumferential movement of the lower punch. Therefore, powder transport by the paddle rotation as well as the filling time emerged as critical determinants. Additionally, all materials showed an increased tendency towards overfilling on the rotary press, possibly due to the higher stress intensity by the paddle wheel.

Lastly, the fill cam geometry also showed a pronounced impact on the filling yield of MCC and LAC. A fill cam suitable for external lubrication gave reduced filling yields on both the rotary press and compaction simulator due to reduced suction filling efficiency. This was explained by the decreased lower punch velocity and residual air inside the die upon entry into the filling zone, acting as a buffer dampening the build-up of differential pressure.

A physics-based calculation of the acting differential pressure using an ODE supported the general experimental observations and offered valuable insights into the impact of machine and fill cam geometry on the strength of suction filling. On this basis, other filling mechanisms, machine geometry and powder properties such as flowability and cohesion should be embedded to translate the calculated suction pressures into a full model for predicting the filling yield on compaction simulators.

The study offers a solid understanding of the complex interplay of machine geometry, process conditions and material properties as well as the underlying mechanisms of die filling on the compaction simulator and rotary press. In the future, the magnitude of the exerted suction pressure should be investigated further by direct pressure measurements. Additionally, a systematic study of the adaption of the process parameters, machine control and feed frame geometry of the compaction simulator should be performed to enable the more accurate reflection of the conditions on the rotary press, aiming to extend the predictive potential of compaction simulation towards die filling.

## CRediT authorship contribution statement

**Ben Kohlhaas:** Writing – review & editing, Writing – original draft, Visualization, Validation, Software, Methodology, Investigation, Formal analysis, Data curation. **Jan Henrik Finke:** Writing – review & editing, Supervision, Resources, Project administration, Funding acquisition, Conceptualization.

## Declaration of competing interest

The authors declare the following financial interests/personal relationships which may be considered as potential competing interests:(Ben Kohlhaas reports financial support was provided by German Research Foundation. Ben Kohlhaas reports equipment, drugs, or supplies was provided by KORSCH AG. Ben Kohlhaas reports equipment, drugs, or supplies was provided by Chemische Fabrik Budenheim KG. Ben Kohlhaas reports equipment, drugs, or supplies was provided by J Rettenmaier and Sons. Ben Kohlhaas reports equipment, drugs, or supplies was provided by MEGGLE GmbH & Co. KG. If there are other authors, they declare that they have no known competing financial interests or personal relationships that could have appeared to influence the work reported in this paper.)

## Data Availability

Data will be made available on request.

## Appendix A Supplementary data

Supplementary material Image 1

## References

[bb0005] Anand A., Curtis J.S., Wassgren C.R., Hancock B.C., Ketterhagen W.R. (2009). Predicting discharge dynamics of wet cohesive particles from a rectangular hopper using the discrete element method (DEM). Chem. Eng. Sci..

[bb0010] Baserinia R., Sinka I.C. (2019). Powder die filling under gravity and suction fill mechanisms. Int. J. Pharmaceut..

[bb0015] Baserinia R., Sinka I.C., Rajniak P. (2016). Vacuum assisted flow initiation in arching powders. Powder Technol..

[bb0020] Darcy H. (1856).

[bb0025] Desbois L., Tchoreloff P., Mazel V. (2019). Influence of the punch speed on the die wall/powder kinematic friction during tableting. J. Pharm. Sci..

[bb0030] Guo Y., Kafui K.D., Wu C.-Y., Thornton C., Seville J.P.K. (2009). A coupled DEM/CFD analysis of the effect of air on powder flow during die filling. AICHE J..

[bb0035] Hairer E., Wanner G. (1996).

[bb0040] Jackson S., Sinka I.C., Cocks A.C.F. (2007). The effect of suction during die fill on a rotary tablet press. Eur. J. Pharm. Biopharm..

[bb0045] Jenike A.W. (1976). Bulletin No. 123; Vol. 53, No. 26, November 1964.

[bb0050] Mazel V., Desbois L., Tchoreloff P. (2019). Influence of the unloading conditions on capping and lamination: Study on a compaction simulator. Int. J. Pharmaceut..

[bb0055] Mehrotra A., Chaudhuri B., Faqih A., Tomassone M.S., Muzzio F.J. (2009). A modeling approach for understanding effects of powder flow properties on tablet weight variability. Powder Technol..

[bb0060] Mendez R., Muzzio F., Velazquez C. (2010). Study of the effects of feed frames on powder blend properties during the filling of tablet press dies. Powder Technol..

[bb0065] Mills L.A., Sinka I.C. (2013). Effect of particle size and density on the die fill of powders. Eur. J. Pharm. Biopharm..

[bb0070] Muskat M. (1937).

[bb0075] Puckhaber D., Finke J.H., David S., Serratoni M., Zafar U., John E., Juhnke M., Kwade A. (2022). Prediction of the impact of lubrication on tablet compactibility. Int. J. Pharmaceut..

[bb0080] Puckhaber D., Kathrin Schomberg A., Kwade A., Henrik Finke J. (2022). A compactibility-based lubricant dispersion model describing the effect of formulation and paddle speed. Int. J. Pharmaceut..

[bb0085] Puckhaber D., Kwade A., Finke J.H. (2023). Investigation of dispersion kinetics of particulate lubricants and their effect on the mechanical strength of MCC tablets. Pharm. Res..

[bb0090] Puckhaber D., Voges A.-L., Rane S., David S., Gururajan B., Finke J.H., Kwade A. (2023). Enhanced multi-component model to consider the lubricant effect on compressibility and compactibility. Eur. J. Pharm. Biopharm..

[bb0095] Puckhaber D., Finke J.H., David S., Gururajan B., Rane S., Kwade A. (2024). Effect of particle size on the dispersion behavior of magnesium stearate blended with microcrystalline cellulose. Int. J. Pharmaceut..

[bb0100] Schneider L., Sinka I.C., Cocks A. (2007). Characterisation of the flow behaviour of pharmaceutical powders using a model die–shoe filling system. Powder Technol..

[bb0105] Schomberg A.K., Kwade A., Finke J.H. (2020). The challenge of die filling in rotary presses-a systematic study of material properties and process parameters. Pharmaceutics.

[bb0110] Schomberg A.K., Kwade A., Finke J.H. (2023). Modeling die filling under gravity for different scales of rotary tablet presses. Powder Technol..

[bb0115] Schomberg A.K., Kwade A., Finke J.H. (2023). Modeling gravity filling of dies on a rotary tablet press. Powder Technol..

[bb0120] Schomberg A.K., Wagner L., Finke J.H., Kwade A. (2025). Development of a model to predict die filling under suction on different rotary tablet presses. Powder Technol..

[bb0125] Schönfeld B., Sundermann J., Keller B.-L., Westedt U., Heinzerling O. (2024). Transformation of ABT-199 nanocrystal suspensions into a redispersible drug product-impact of vacuum drum drying, spray drying and tableting on re-nanodispersibility. Pharmaceutics.

[bb0130] Sinka I.C., Schneider L.C.R., Cocks A.C.F. (2004). Measurement of the flow properties of powders with special reference to die fill. Int. J. Pharmaceut..

[bb0135] Sinka I.C., Motazedian F., Cocks A., Pitt K.G. (2009). The effect of processing parameters on pharmaceutical tablet properties. Powder Technol..

[bb0140] Sun C.C. (2015). Dependence of ejection force on tableting speed—a compaction simulation study. Powder Technol..

[bb0145] Tang X., Zakhvatayeva A., Zhang L., Wu Z.-F., Sun P., Wu C.-Y. (2020). Flow behaviour of pharmaceutical powders during rotary die filling with a paddle feeder. Int. J. Pharmaceut..

[bb0150] Virtanen P., Gommers R., Oliphant T.E., Haberland M., Reddy T., Cournapeau D., Burovski E., Peterson P., Weckesser W., Bright J., van der Walt S.J., Brett M., Wilson J., Millman K.J., Mayorov N., Nelson A.R.J., Jones E., Kern R., Larson E., Carey C.J., Polat İ., Feng Y., Moore E.W., VanderPlas J., Laxalde D., Perktold J., Cimrman R., Henriksen I., Quintero E.A., Harris C.R., Archibald A.M., Ribeiro A.H., Pedregosa F., van Mulbregt P. (2020). SciPy 1.0: fundamental algorithms for scientific computing in Python. Nat. Methods.

[bb0155] Wang J.J., Guillot M.A., Bateman S.D., Morris K.R. (2004). Modeling of adhesion in tablet compression. II. Compaction studies using a compaction simulator and an instrumented tablet press. J. Pharm. Sci..

[bb0160] Wu C.-Y., Guo Y. (2012). Numerical modelling of suction filling using DEM/CFD. Chem. Eng. Sci..

[bb0165] Wu C.-Y., Dihoru L., Cocks A.C. (2003). The flow of powder into simple and stepped dies. Powder Technol..

[bb0170] Wünsch I., Friesen I., Puckhaber D., Schlegel T., Finke J.H. (2020). Scaling Tableting Processes from Compaction Simulator to Rotary Presses-mind the Sub-Processes. Pharmaceutics.

[bb0175] Zakhvatayeva A., Zhong W., Makroo H.A., Hare C., Wu C.Y. (2018). An experimental study of die filling of pharmaceutical powders using a rotary die filling system. Int. J. Pharmaceut..

[bb0180] Zakhvatayeva A., Hare C., Wu C.Y. (2019). Suction filling of pharmaceutical powders. Powder Technol..

