# Supplementary material for: Investigation of differences in mechanisms of die filling between a compaction simulator and a rotary press
Source: Int J Pharm X. 2025 Sep 23;10:100405. doi: 10.1016/j.ijpx.2025.100405 (PMC12549384; doi:10.1016/j.ijpx.2025.100405)
Supplement: Supplementary file 1 — Supplementary material [file mmc1.docx]

## **Parameters for numerical calculation**

| **parameter** | **input value** |
| --- | --- |
| $R$ | $8.314 \frac{\text{J}}{\text{mol⋅K}}$ |
| $T$ | $293.15 \text{K}$ |
| $p_{\text{atm}}$ | $101325 \text{Pa}$ |
| $\rho_{\text{air}}$ | $1.204 \frac{\text{kg}}{\text{m}^{\text{3}}}$ |
| $\eta_{\text{air}}$ | $1.825\cdot{10}^{-5}\text{ }\text{Pa⋅s}$ |
| $M_{\text{air}}$ | $0.02849 \frac{\text{kg}}{\text{mol}}$ |
| $h_{0}$ (conventional fill cam) | $0 \text{mm}$ |
| $h_{0}$ (stearate fill cam) | $4.7 \text{mm}$ |
| punch travelling distance (conventional fill cam) | $16 \text{mm}$ |
| punch travelling distance (stearate fill cam) | $11.3 \text{mm}$ |

Table 1 - Parameters for numerical calculation

## **Regression functions stationary velocity– Rotary press**

### **conventional fill cam**

| $v=2.13333 \frac{\text{mm}\cdot\text{min}}{\text{s}}\cdot n_{\text{t}}+4.26326\cdot{10}^{-14} \frac{\text{mm}}{\text{s}}$ | (1) |
| --- | --- |

### **stearate fill cam**

| $v=1.5064 \frac{\text{mm}\cdot\text{min}}{\text{s}}\cdot n_{\text{t}}-1.4209\cdot{10}^{-14} \frac{\text{mm}}{\text{s}}$ | (2) |
| --- | --- |

## **Regression functions for punch acceleration, stationary velocity and deceleration – Compaction simulator**

### **conventional fill cam**

1. Acceleration

| $a_{\text{a}}=0.53529 \frac{\text{mm}\cdot\text{mi}\text{n}^{\text{2}}}{\text{s}^{\text{2}}}\cdot n_{\text{t}}^{2}+50.70183 \frac{\text{mm}\cdot\text{min}}{\text{s}^{\text{2}}}\cdot n_{\text{t}}+155.90106 \frac{\text{mm}}{\text{s}^{\text{2}}}$ | (3) |
| --- | --- |

1. Stationary velocity

| $v=0.01628 \frac{\text{mm}\cdot\text{mi}\text{n}^{\text{2}}}{\text{s}}\cdot n_{\text{t}}^{2}+1.87489 \frac{\text{mm}\cdot\text{min}}{\text{s}}\cdot n_{\text{t}}+2.85626 \frac{\text{mm}}{\text{s}}$ | (4) |
| --- | --- |

1. Deceleration

| $a_{\text{d}}=0.07373 \frac{\text{mm}\cdot\text{mi}\text{n}^{\text{2}}}{\text{s}^{\text{2}}}\cdot n_{\text{t}}^{2}+84.43344 \frac{\text{mm}\cdot\text{min}}{\text{s}^{\text{2}}}\cdot n_{\text{t}}-487.82303 \frac{\text{mm}}{\text{s}^{\text{2}}}$ | (5) |
| --- | --- |

### **stearate fill cam**

1. Acceleration

| $a_{\text{a}}=0.42751 \frac{\text{mm}\cdot\text{mi}\text{n}^{\text{2}}}{\text{s}^{\text{2}}}\cdot n_{\text{t}}^{2}+30.59925 \frac{\text{mm}\cdot\text{min}}{\text{s}^{\text{2}}}\cdot n_{\text{t}}+241.11626 \frac{\text{mm}}{\text{s}^{\text{2}}}$ | (6) |
| --- | --- |

1. Stationary velocity

| $v=0.01205 \frac{\text{mm}\cdot\text{mi}\text{n}^{\text{2}}}{\text{s}}\cdot n_{\text{t}}^{2}+1.28219 \frac{\text{mm}\cdot\text{min}}{\text{s}}\cdot n_{\text{t}}+2.69603 \frac{\text{mm}}{\text{s}}$ | (7) |
| --- | --- |

1. Deceleration

| $a_{\text{d}}=0.53885 \frac{\text{mm}\cdot\text{mi}\text{n}^{\text{2}}}{\text{s}^{\text{2}}}\cdot n_{\text{t}}^{2}+19.79705 \frac{\text{mm}\cdot\text{min}}{\text{s}^{\text{2}}}\cdot n_{\text{t}}+384.52726 \frac{\text{mm}}{\text{s}^{\text{2}}}$ | (8) |
| --- | --- |

## **Die filling experiments - tablet weight variation**

Figure S.1 - Tablet weight variation for DCP: a) conventional fill cam on rotary press, b) stearate fill cam on rotary press, c) conventional fill camp on compaction simulator, d) stearate fill cam on compaction simulator

Figure S.2 - Tablet weight variation for MCC: a) conventional fill cam on rotary press, b) stearate fill cam on rotary press, c) conventional fill camp on compaction simulator, d) stearate fill cam on compaction simulator

Figure S.3 - Tablet weight variation for LAC: a) conventional fill cam on rotary press, b) stearate fill cam on rotary press, c) conventional fill camp on compaction simulator, d) stearate fill cam on compaction simulator
